# Alcohol screening for older adults in an acute general hospital: FAST *v.* MAST-G assessments

**DOI:** 10.1192/pb.bp.114.049734

**Published:** 2016-04

**Authors:** Rachel Knightly, George Tadros, Juhi Sharma, Peter Duffield, Emma Carnall, Jacqui Fisher, Shaza Salman

**Affiliations:** 1 Heart of England NHS Foundation Trust, Heartlands Hospital, Birmingham; 2 Birmingham and Solihull Mental Health Foundation Trust, City Hospital, Birmingham; 3 Aquarius, Birmingham

## Abstract

**Aims and method** Documented prevalence of alcohol misuse among older adult patients at Birmingham Heartlands Hospital is significantly lower than the national prevalence. We aimed to evaluate our alcohol misuse screening protocol for older adults to identify possible shortcomings. Hospital protocol is to screen all adults for alcohol misuse in the accident and emergency (A&E) department using the Fast Alcohol Screening Test (FAST). One hundred consecutive consenting in-patients aged 65-94 admitted via A&E subsequently undertook an additional alcohol screening test (Michigan Alcoholism Screening Test-Geriatric version; MAST-G). Results of the two tests were compared.

**Results** FAST screening was completed for 71 patients and none were FAST-positive for alcohol misuse, yet using MAST-G, 18 patients scored positively for alcohol misuse. FAST screening failed to identify 8 patients with a documented history of alcohol misuse.

**Clinical implications** Older adult alcohol misuse prevalence is significantly underreported using FAST. Screening older adults for alcohol problems requires a different approach to screening the general population.

In 2010, 20% of men and 9% of women aged 65 or over in the UK were misusing alcohol (for men this means drinking >21 units/week, for women drinking >14 units/week).^1^ Alcohol misuse prevalence in this population is increasing.^2,3^ With the rising number of older adults living in the UK,^4^ the prevalence of substance use problems among this age group is predicted to more than double between 2001 and 2020.^5-7^ Despite the national figures being much higher, screening at Birmingham Heartlands tertiary care hospital has historically identified only a 1% prevalence of alcohol misuse among in-patients aged 65 or over.

Metabolic and physiological changes, and long-term polypharmacy, put older people at high risk of adverse physical effects of alcohol misuse, including peptic ulcer disease and falls.^8-12^ Alcohol misuse in the elderly can often be misdiagnosed or undetected:^13^ reasons include different presentations compared with younger adults, stigma and difficulties screening older adults.^14,15^ Screening tests, including the Michigan Alcoholism Screening Test – Geriatric version (MAST-G) have been developed to overcome these difficulties. Compared with the Fast Alcohol Screening Test (FAST), a robust screening tool for the general population,^16^ MAST-G has higher sensitivity for alcohol misuse in older adults.^17^

We hypothesised that there is no difference in using FAST or MAST-G to identify older people with increasing alcohol intake in an acute hospital setting. We aimed to conduct further screening and medical history review to better understand the pattern of alcohol misuse in the older adult population in our hospital and compare the true prevalence of alcohol misuse to that identified by our current screening protocol.

## Method

### Participants

Consecutive in-patients aged 65 or over admitted to the acute medical unit via the accident and emergency (A&E) department were identified. Patients were excluded from participation if they were medically unfit for interview, if they were acutely confused, or if communication in English was difficult. Eligible patients were invited to undertake the MAST-G alcoholism screening test. The test consists of 24 questions about alcohol use habits with yes/no responses. A score of five or more questions answered positively indicates alcohol misuse (older adults sensitivity 94.9% and specificity 77.8%).^17^ The first 100 eligible patients who gave verbal consent and who completed MAST-G were included in the service evaluation; 7 eligible patients did not give their consent to participate.

### Procedure

MAST-G was completed either by patients themselves or by a member of staff reading questions aloud and recording patient responses. Participating patients' A&E notes were retrospectively examined and their FAST score documented; the MAST-G scores and the A&E FAST scores were compared. History of alcohol misuse was identified from patients' records from previous hospital admissions.

### Analysis

Results were analysed using SPSS (version 19 for Windows). Frequency data are reported as *n* (%). Non-parametric data were analysed using related-samples Wilcoxon signed rank test, Fisher's exact test and Spearman's rank correlation coefficient. Two-tailed *P*-values are given: a threshold of *P*<0.05 was used to determine statistical significance.

## Results

### Population

One hundred older adults completed a MAST-G questionnaire, answering all 24 questions. Their median age was 79 years (interquartile range 73-86, range 65-94, *n* = 100). The majority (61%) were female. The most commonly documented reasons for patients' admission to the acute medical unit were fall (*n* = 14), shortness of breath (*n* = 10) and chest pain (*n* = 9).

### FAST and MAST-G scores among different patient groups

Of the 100 participants, 71 (71%) had a FAST score documented in A&E (Table 1). In none of these was the FAST score positive. In contrast, 18 participants (18%) subsequently scored positively for alcohol misuse using MAST-G. The difference in patients' scores between the two tests was statistically significant (*P*<0.0001). Among the 18 patients with positive MAST-G scores, 12 had scored negatively for alcohol misuse on FAST, 4 patients had been unable to answer FAST in A&E and 2 patients were not asked FAST screening questions.

**Table 1:** Fast Alcohol Screening Test (FAST) outcomes for participants who scored positively and negatively on the Michigan Alcoholism Screening Test – Geriatric version (MAST-G); (*P*<0.0001)

	MAST-G		
FAST	Positive	Negative	Total
Positive	0	0	0

Negative	12	59	71

No result	6	23	29

**Fig. 1:**
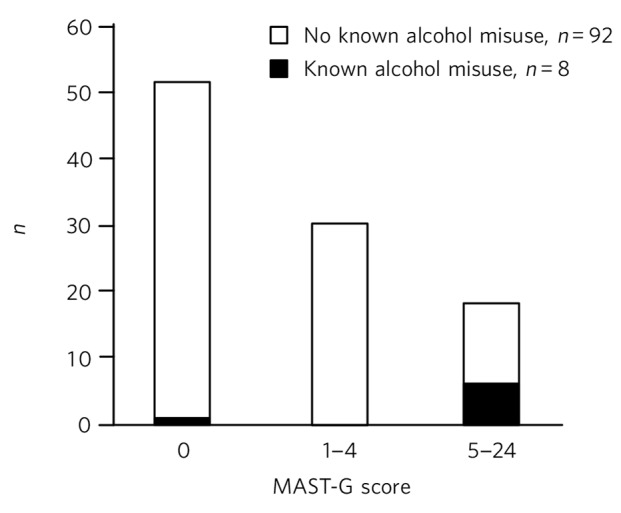
A MAST-G histogram for the 100 participants. A score of ⩾5 indicates alcohol misuse.

Hazardous or harmful alcohol misuse was documented in the medical records of eight patients. Six of these patients had answered FAST questions in A&E (one patient was unable to answer, one patient not asked), but none scored positively on FAST. Seven out of the eight patients (87.5%) subsequently scored positively on MAST-G (Fig. 1). There was a significant association between those older adults who had a history of alcohol misuse and those who scored positively on MAST-G (*P*<0.0001).

Men were significantly more likely to score positively on MAST-G than women (12/29 men and 6/61 women scored positively; *P* = 0.015). No correlation was observed between MAST-G score and patient age (Spearman's rank correlation coefficient −0.164; *P* = 0.1).

The question most frequently answered ‘yes’ by participants with a history of alcohol misuse was ‘Does having a drink help you sleep?’ – 75% answered ‘yes’ compared with 22.8% of patients with no history of alcohol misuse (Table 2).

**Table 2:** The six Michigan Alcoholism Screening Test – Geriatric version (MAST-G) questions to which older adults with a history of alcohol misuse most frequently answered ‘yes’

Question	Patients with a history of alcohol misuse answering ‘yes’ (*n* = 8), *n* (%)	Patients with no history of alcohol misuse answering ‘yes’ (*n* = 92), *n* (%)
1 Does having a drink help you sleep?	6 (75)	21 (22.8)

2 Does alcohol sometimes make it hard for you to remember parts of the day or night?	4 (50)	2 (2.2)

3 Do you have rules for yourself that you won't drink before a certain time of the day?	4 (50)	10 (10.9)

4 Have you ever been concerned that drinking might be harmful to your health?	4 (50)	12 (13.0)

5 In general, would you prefer to have a few drinks at home rather than go out to social events?	4 (50)	17 (18.5)

6 Has a doctor or nurse ever said they were worried or concerned about your drinking?	4 (50)	2 (2.2)

## Discussion

### Main findings

The current hospital screening protocol (FAST) did not identify alcohol misuse in any of the 100 participating older adults, including 8 patients with a documented history of alcohol misuse. On further screening, 18% of the same older adults were identified as misusing alcohol using MAST-G; this proportion is more consistent with previously reported national figures.^1^ This service evaluation highlights a difference between the number of older adult patients identified as misusing alcohol using our standard practice (FAST in A&E) and those identified after admission using an alternative screening test.

### Targets for alcohol screening in adults

The National Institute for Health and Care Excellence recommends that National Health Service (NHS) professionals should ‘routinely carry out alcohol screening as an integral part of practice’.^18^ Locally, as part of the Making Every Contact Count campaign, the Heart of England NHS Foundation Trust mandated that 50% of A&E attendees be screened for alcohol misuse and applied a financial penalty if this target was not met. This target was met in our subpopulation (71% were screened) but our results suggest that screening did not contribute to an increased awareness of, or service provision for, older adults admitted with alcohol misuse issues during this service evaluation.

### An aging population with increasing alcohol misuse

Increasing alcohol misuse among older adults in the UK leads to health and social problems, as well as increasing the risk of accidents necessitating hospital admission and causing significant mortality.^2,3,19,20^ Both acute and long-term complications of alcohol withdrawal are more severe in older adults.^21,22^ The Royal College of Psychiatrists' recommendation to reduce the ‘safe limit’ for alcohol intake from 21 units per week for men and 14 units per week for women to 11 units per week for older adults reflects the increased risks of alcohol misuse in this population.^21^

This trust has recognised the increased prevalence and risks of alcohol misuse among older adults, but our results compared with historical annual prevalence figures (documented as 1% in this trust) highlight an ongoing failure to recognise alcohol misuse in this population, despite the achievement of local screening targets.

### Older adults: a difficult population to screen

Other researchers have found older adults a difficult population to screen for alcohol misuse, depression and delirium,^23-25^ possibly due to stigma attributed to alcohol by adults in this population^26^ or unwillingness to disclose information perceived to contribute little to an acute medical or surgical assessment. However, results from our evaluation show that most eligible older adults were willing to discuss alcohol misuse when invited to take part in the evaluation: only seven patients refused consent. Correlation with documented alcohol misuse indicates that those participants who undertook MAST-G were, on the whole, open about their drinking habits. It is therefore worth considering what other factors could have contributed to our findings.

By investigating staff attitudes to older adult alcohol misuse, previous studies have found hospital staff to have less suspicion and more tolerance of alcohol misuse in older adults than the working-age population,^27^ resulting in low levels of detection and low levels of referral for recognised problems.^28^ In addition, it may in fact be harder for staff to detect alcohol misuse in older adults than it is in working-age adults^23^ – notably, the pattern of alcohol misuse can be different in older adults.^29^ MAST-G was developed to account for these differences, as FAST questions about meeting work responsibilities and concerns voiced by professionals or family members may be less relevant to older adults. MAST-G lifestyle-specific questions about daytime somnolence and social withdrawal aim to maximise sensitivity to typical presentations of alcohol misuse in older adults.

Another reason for the difference between FAST results in A&E and MAST-G results in the acute medical unit could have been the different physical and temporal environments in which screening took place. FAST screening contributes to a battery of questions and investigations in A&E, where patients may feel stressed or time-pressured, and healthcare staff have a large number of clinical decisions to prioritise. Previous studies have suggested that the busy A&E environment could negatively affect clinicians' screening methods and documentation.^27^ Indeed, response rates were lower for FAST in A&E compared with MAST-G in the acute medical unit: 13/100 (13%) of the study population refused to answer FAST (and 14 were unable to answer) compared with 7/107 (6.5%) eligible patients who refused MAST-G. It is possible that the slower paced questioning afforded by MAST-G in a more relaxed environment enabled patients to consider their answers more carefully than those given during FAST, and allowed clinicians more time to engage with participants' responses. If this is the case, and patients' environment affects their willingness to undergo screening, we should consider even more carefully our policy of undertaking screening of older adults in A&E.

### Practice implications highlighted by this service evaluation

Throughout patient interactions as part of this service evaluation it has become clear that many older adults do not recognise or report their significant alcohol misuse as harmful. It falls to healthcare staff to be opportunistic in their direct questioning about alcohol misuse and to give advice appropriately. Lack of opportunistic diagnosis and intervention from healthcare professionals not only inhibits optimal care during hospital admission, but denies patients the chance to make informed decisions about their long-term health and access to out-patient services. A proactive approach appears key to our discussions about alcohol use.

This evaluation suggests that our current process is grossly underestimating true prevalence of older adult alcohol misuse. FAST has a sensitivity of 93% for alcohol misuse in the general population, but its sensitivity in older adults is less certain. The current evaluation has not identified whether it is the screening tool itself, the environment in which patients are screened, or the clinician-patient interaction during screening which has lowered our sensitivity for alcohol misuse so significantly. Importantly for our future practice, although our results indicate that MAST-G may be a more sensitive screening tool for our population, the time taken to complete its 24 questions makes it an unattractive screening test for A&E. In identifying problems with our current screening system, this service evaluation has not suggested a comparable alternative. Further prospective work is needed to determine the best way for us to accurately identify older adult alcohol misuse both efficiently and sensitively.

Early opportunistic diagnosis of alcohol misuse has clear benefits for both primary and secondary healthcare provision; undiagnosed alcohol misuse has significant social and economic implications, as well as an impact on physical health.^21^ Given the findings of this service evaluation, research into the sensitivity of alcohol screening of older adults in the community will complement our work.

## Limitations

At this hospital every adult A&E attendee considered fit to answer screening questions is offered 4-question FAST screening, whereas only eligible medical in-patients willing to take part in this evaluation undertook the longer 24-question MAST-G and formed part of our analysis. The first limitation of this approach is that we have not evaluated directly comparable tests, but nor did we aim to do so. Our aim was to identify shortcomings in our current screening method in light of putative inconsistencies with national data. We have not identified an improved method of screening as a result of this study. Rather, we have confirmed that our current procedure is failing to identify important information which patients are, in fact, willing to disclose in an alternative environment.

The second limitation resulting from our selection of in-patient participants is that our sample population does not fully represent the population of older adults attending A&E or, indeed, the wider population in the community. It is possible that alcohol misuse could have been a precipitating factor for medical admission among interviewees, leading to a biased sample and skewed results. In addition, we did not ask individuals' reasons for withholding consent to complete MAST-G (seven eligible patients refused consent). Selection bias may have increased if participants unwilling to discuss ongoing alcohol misuse withheld consent to avoid detection and intervention.

When considering the true prevalence of alcohol misuse in this population, clinical history of alcohol misuse was taken from patients' recent medical notes. As such, any undocumented alcohol misuse was counted as absence of alcohol misuse, leading to a potential under-estimation of misuse prevalence. This limitation does not negate our findings; rather, it adds weight to our need for an effective screening protocol.

As a result of the screening protocol used in our typically busy general hospital A&E department, alcohol misuse went unidentified in a population with both known (documented) and freely volunteered (via MAST-G) alcohol misuse. Studies comparing FAST with MAST-G in the same temporal and physical setting will complement this initial evaluation, allowing us to understand why we are failing to identify misuse. Analysis of the reasons for discrepancies in the results from those two screening tools will aid our understanding of how to sensitively and efficiently screen for alcohol misuse in our older adult population.
